# MERTK in the rat trigeminal system: a potential novel target for cluster headache?

**DOI:** 10.1186/s10194-024-01791-6

**Published:** 2024-05-23

**Authors:** Jacob C. A. Edvinsson, Caroline Ran, Felicia Jennysdotter Olofsgård, Anna Steinberg, Lars Edvinsson, Andrea Carmine Belin

**Affiliations:** 1 Department of Internal Medicine, Lund University, Sölvegatan 19, Lund, 22184 Sweden; 2 Centre for Cluster Headache, Department of Neuroscience, Karolinska Institutet, Stockholm, 17177 Sweden; 3 Department of Clinical Neuroscience, Karolinska Institutet, Stockholm, 17177 Sweden; 4 Department of Neurology, Karolinska University Hospital, Stockholm, 17176 Sweden

**Keywords:** Trigeminal system, MER proto-oncogene tyrosine kinase, Migraine, GWAS, Galectin-3

## Abstract

The trigeminal system is key to the pathophysiology of migraine and cluster headache, two primary headache disorders that share many features. Recently, MER proto-oncogene tyrosine kinase (*MERTK*), a cell surface receptor, was strongly associated with cluster headache through genetic studies. Further, the MERTK ligand galectin-3 has been found to be elevated in serum of migraine patients. In this study, MERTK and MERTK ligands were investigated in key tissue to better understand their potential implication in the pathophysiology of primary headache disorders. Immunohistochemistry was used to map MERTK and galectin-3 expression in rat trigeminal ganglia. RT-qPCR was used to assess *MERTK* gene expression in blood, and ELISA immunoassays were used for MERTK ligand quantification in serum from study participants with and without cluster headache. *MERTK* gene expression was elevated in blood samples from study participants with cluster headache compared to controls. In addition, MERTK ligand galectin-3 was found at increased concentration in the serum of study participants with cluster headache, whereas the levels of MERTK ligands growth arrest specific 6 and protein S unaffected. MERTK and galectin-3 were both expressed in rat trigeminal ganglia. Galectin-3 was primarily localized in smaller neurons and to a lesser extent in C-fibres, while MERTK was found in satellite glia cells and in the outer membrane of Schwann cells. Interestingly, a strong MERTK signal was found specifically in the region proximal to the nodes of Ranvier. The overexpression of MERTK and galectin-3 in tissue from study participants with cluster headache, as well as the presence of MERTK in rat peripheral satellite glia cells and Schwann cells in the trigeminal ganglia, further highlights MERTK signalling as an interesting potential future therapeutic target in primary headache.

**Graphical Abstract:**

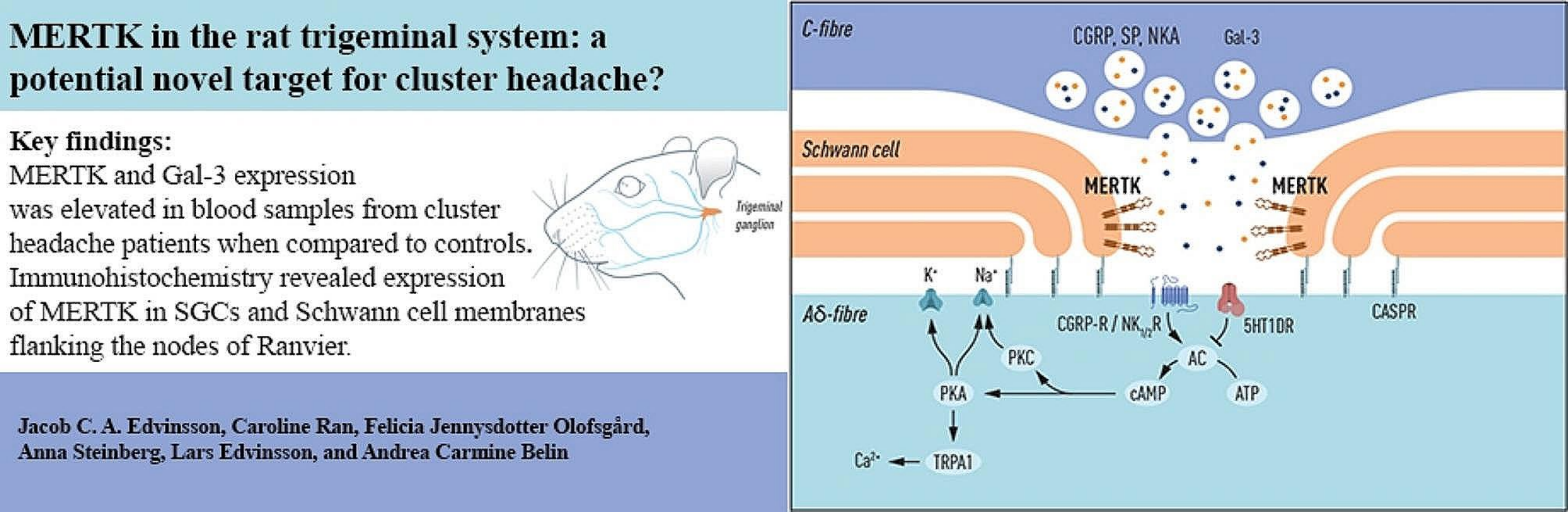

**Supplementary Information:**

The online version contains supplementary material available at 10.1186/s10194-024-01791-6.

## Introduction

Migraine and cluster headache (CH) are some of the most widespread and disabling primary headache conditions in the world [1]. Migraine is considered to afflict approximately 15% of the global population, while CH is considerably rarer, with an estimated prevalence of 0.1% [2]. Though the two headache disorders manifests differently clinically, migraine and CH have several similarities such as unilateral pain and responsiveness to triptans and, to a certain extent, calcitonin gene-related peptide (CGRP) monoclonal antibody intervention [3]. While migraine has been shown to be more prevalent in females compared to males (at a 3:1 ratio), the opposite relationship has been suggested to apply to CH [4].

The most important clinical hallmark of CH is the extremely strong pain experienced during active periods (known as bouts), lasting weeks or months, which is mediated by the trigeminal system [5]. CH attacks are considered frequent but relatively short (∼ 15 min to 3 h), which differs from migraine attacks which can last for several days. Another interesting difference between the two headaches is that CH is suggested to be linked to the circadian rhythm as attacks often occur during the night [6].

Although the precise mechanisms underlying both conditions are yet to be comprehensively elucidated, the trigeminal system serves as a pathophysiological pathway applicable to both disorders. First order neurons arising from the trigeminal ganglion (TG), which innervates the skin, dura, and vasculature of the cranium, project to the trigeminal nucleus caudalis (TNC) [7] in the brainstem, synapsing with ascending second order neurons. The pain signal is then propagated to the third order neurons in the thalamus from where it is finally relayed to and interpreted by the cortical pain centers [8].

The nociceptive trigeminal neurons can be categorized into two types, small to medium sized neurons that form C-fibres, and medium to large sized neurons forming primarily Aδ-fibres [9]. It is widely accepted that the trigeminal C-fibres express CGRP and Aδ-fibres express calcitonin receptor-like receptor/receptor activity-modifying protein 1 (CLR/RAMP1, together forming the CGRP receptor) which have key roles in primary headaches [9]. In the TG the neurons are enveloped by satellite glial cells (SGCs) connected via gap junctions, which communicate intimately with the closely situated neurons [9]. It is suggested that the SGCs may modify the activity of the neurons via crosstalk mechanisms and are involved in sterile inflammatory response [10].

The recent findings in two parallel *Genome-Wide Association* Studies (GWAS), identifying risk loci for CH, highlighted a strong association with a locus at chromosome 2q13 which harbours the MER proto-oncogene, tyrosine kinase (*MERTK*) gene [11, 12]. This association was further confirmed in another GWAS, as well as in a meta-GWAS [13, 14]. Significant genetic markers within these loci were linked to several expression quantitative trait loci (eQTLs) which potentially affect the gene expression of *MERTK* [11–14]. The *MERTK* gene encodes a receptor tyrosine kinase of the TAM (Tyro3, Axl, MERTK) family, which is involved in phagocytosis, particularly of apoptotic cells. It is reported to have an important role in neuroinflammation and to be highly expressed in microglia, astrocytes, and macrophages. *MER* is the orthologous murine gene of human *MERTK*, and MER-deficient mice have impaired phagocytosis of the retinal pigment epithelium associated with severe retinal degeneration [15]. They also exhibit an aggregation of apoptotic cells in neurogenic regions of the central nervous system [16]. However, it should be noted that the phenotype of MER-deficient mice may be a result of other changes in the genome of these mice, e.g. altered expression of Tyro3 [17]. A genetic association has been found between single nucleotide polymorphisms (SNPs) in *MERTK* and multiple sclerosis [18]. A study on MER-deficient mice showed that these animals have impaired remyelination after induced demyelination and suggest a mechanism involving MERTK-dependent microglial activation and clearance of myelin debris preceding a successful remyelination [19]. Other pathologies genetically linked to *MERTK* are retinal dystrophy and liver fibrosis [20, 21]. Furthermore, since its discovery MERTK has been linked to the pathogenesis of cancer [22] in which MERTK has been observed to be overly or ectopically expressed [23]. This overexpression has been suggested to result in the activation of several canonical oncogenic signalling pathways such as the mitogen-activated protein kinase (MAPK) and phosphoinositide 3-kinase (PI3K) pathways [24].

There are several known ligands for MERTK including galectin-3 (Gal-3), protein S (PROS1) and growth arrest specific 6 (GAS6). They act either as free proteins or attached to apoptotic cells during MERTK-mediated clearance of apoptotic cells (efferocytosis) [25–27]. Gal-3 is an important pro-inflammatory mediator associated with acute and chronic inflammation [28, 29]. Furthermore, Gal-3 is reported to have a key role in neuroinflammation and is the only MERTK ligand which has so far been implicated in headache pathology [30, 31]. In a study investigating Gal-3 serum levels in migraine, Gal-3 levels were significantly higher in samples from study participants with migraine compared to controls. However, there was no difference in Gal-3 levels during the attacks and the interictal period within the migraine group [32]. Two further recent studies have confirmed elevated Gal-3 serum levels in migraine patients during migraine attacks when compared to healthy controls, but no difference was observed between the cases with and without aura [33, 34].

The aim of this study was to examine and identify the potential role of MERTK in CH, and its possible relation to CGRP signalling which is considered to be affected in many primary headaches. To characterize MERTK as a potential player in headache pathophysiology we have investigated protein expression in neuronal tissue from rats and further compared *MERTK* gene expression and levels of MERTK ligands in blood from study participants with a validated CH diagnosis to blood from control subjects.

## Materials and methods

### Animals

All animal procedures in this study followed the guidelines of the European Communities Council (86/609/ECC) and were approved by the Regional Ethical Committee on Animal Research, Malmö/Lund, Sweden (M17-15).

### Human tissue

The study on human tissue was approved by the Swedish Ethical Review Authority in Stockholm, Sweden (217/02  and 2014/656 − 31/4). Cluster headache patients (*n* = 27) and neurologically healthy control individuals (*n* = 30) were recruited at the Neurology clinic, Karolinska University Hospital, Stockholm, Sweden. Validation of CH diagnosis was made according to the International Classification of Headache Disorders 3rd edition (ICHD) by a neurologist, co-author A.S. [1]. All participants signed an informed consent. A venous blood sample was drawn from participants using either PAX tubes for RNA preparation (Becton Dickinson Sweden AB, Stockholm, Sweden), or regular EDTA tubes for serum extraction. PAX tubes were stored at -20°C until use. Serum was isolated from EDTA tubes and stored at -80°C until use. Blood samples from study participants with CH were extracted during active bout or in remission period (see details in Table 1).

**Table 1:** Details of the cohort

Disease status	Analysis	Subjects (n)	Episodic/chronic subtype (n)	Female sex (n)	Average age (years)	In active bout (n)
Cluster headache	Rt-qPCR	16	16/0	12.5% (2)	48.4	37.5% (6)
	ELISA	11	10/1	45.5% (5)	50.3	54.5% (6)
Healthy control	Rt-qPCR	21	NA	38.1% (8)	39.0	NA
	ELISA	9	NA	0% (0)	44.9	NA

NA: Not applicable

### Animal tissue preparation

Tissue from adult male Wistar rats (*n* = 6, weight 250–300 g) was used for immunohistochemistry. Animals were anaesthetized with CO_2_ and subsequently decapitated. TGs were carefully dissected and submerged in 4% paraformaldehyde solved in phosphate buffer saline (PBS). The TGs were fixed in this solution for 2–4 h at room temperature. The tissues were submerged in Sörensen’s phosphate buffer (pH 7.2) containing 10% and 25% sucrose overnight. Lastly, the tissues were embedded in a gelatin medium (30% egg albumin, 3% gelatin), cryosectioned (10 μm) and collected on microscope slides (Superfrost, ThermoFisher). Samples were stored at -20 °C until use.

### Immunohistochemistry

After thawing at room temperature, the sections were permeabilized by submerging the slides in PBS containing 0.25% Triton X-100 (PBST) for 2 × 15 min. Primary antibodies (Supplementary Table 1a) diluted in PBS-T containing 1% bovine serum albumin (BSA, Sigma) were applied to the sections that were then incubated at + 4 °C overnight. Subsequently, the excess primary antibodies were rinsed by washing the sections in PBST for 2 × 15 min. Secondary antibodies (Supplementary Table 1b), corresponding to the primary antibodies’ species, were applied to the sections and incubated in a dark room, for one hour at room temperature. Excess secondary antibodies were rinsed by washing with PBST for 2 × 15 min. Finally, slides were mounted with anti-fading medium containing 4’,6-diamidino-2-phenylindole (DAPI) (Vectashield, Vector Laboratories, Burlingame CA, USA). For double immunohistochemistry the procedure was repeated before mounting. Negative controls were included by omitting the primary antibody to evaluate auto-fluorescence and non-specific secondary antibody binding levels.

The sections were analyzed using an epifluorescence microscope (Nikon 80i; Tokyo, Japan) at the appropriate wavelengths and photographed with an attached Nikon DS-2Mv camera. Images were processed into figure montages using Adobe Photoshop CC 2023 (Adobe Systems, Mountain View, CA, USA).

### Rt-qPCR

RNA preparation from blood collected in PAX tubes was performed using PAXgene Blood RNA kit according to the manufacturer’s instructions (QIAGEN). cDNA was subsequently prepared from 500 ng RNA using Quantitect Reverse Transcription Kit (QIAGEN) according to standard protocols.

Gene expression was analyzed by reverse-transcription quantitative real-time PCR (Rt-qPCR) run on a QuantStudio™ 5 real-time system (Thermo Fisher) with TaqMan® reagents. The qPCR cycler was programmed to run an initiation step 50 °C for 2 min and 95 °C for 2 min followed by 40 cycles of denaturation at 95 °C for 1 s and annealing/extension at 60 °C for 20 s. We used TaqMan® fast advanced master mix at a concentration of 1X and 200 ng cDNA for each reaction, together with TaqMan® gene expression assays HS00427620_ml (TATA-box binding protein, *TBP*), HS00914057_ml (Importin 8, *IPO8*) and HS01031979_ml (*MERTK*) at a concentration of 1X (Thermo Fisher). *MERTK* expression levels were normalized to the two housekeeping genes, *TBP* and *IPO8*, and then to a randomly selected control sample to obtain relative gene expression levels.

### ELISA

Gal-3, GAS6 and PROS1 concentrations were measured in serum samples from CH patients and control individuals by means of enzyme linked immunosorbent assays (ELISA). We used immunosorbent Assay BMS279-4 for Gal-3 (analytical sensitivity: 0.29 ng/ml), BMS2291 for GAS6 (analytical sensitivity: 0.65 pg/ml), and EEL043 for PROS1 (analytical sensitivity: 0.19 ng/ml) (Invitrogen). Assays were performed according to the recommended protocols, including standard curves for quantitation. Plates were read at 450 nm using a Multiscan FC plate reader (Thermo Scientific). All samples were run in triplicates, background absorbance was subtracted from each measurement, whereafter average absorbance for each sample was calculated and the concentration was obtained from the standard curve considering the sample dilutions that were made during the experiment.

### Statistical analysis

The presence of outliers in the data was evaluated with Grubb’s test (GraphPad QuickCalcs, GraphPad Software, Boston, US). No outliers were detected in gene expression data, max sample Z-value = 2.79, as compared to a critical Z-value of 3.00 with a significance level of α = 0.05 (two sided). One outlier was detected in data from ELISAs, max sample Z-value = 2.93, as compared to a critical Z-value of 2.71 with a significance level of α = 0.05 (two sided) leading to the removal of one control sample in the Gal-3-analysis. Data was analyzed and visualized using Rv4.1.1 and R Studio v.2021.09.0 [35]. Shapiro-Wilk test was used to assess the normal distribution of the data. Gene expression data was log2-transformed before group analysis. The Student’s t-test (two tailed p-values) was used for group comparison of normal data, The Wilcoxon rank-sum test was used for non-normal data. Significance level was set to α = 0.05. Correction for multiple testing was applied when analyzing MERTK ligand concentrations.

## Results

### Immunohistochemistry

**Fig. 1:**
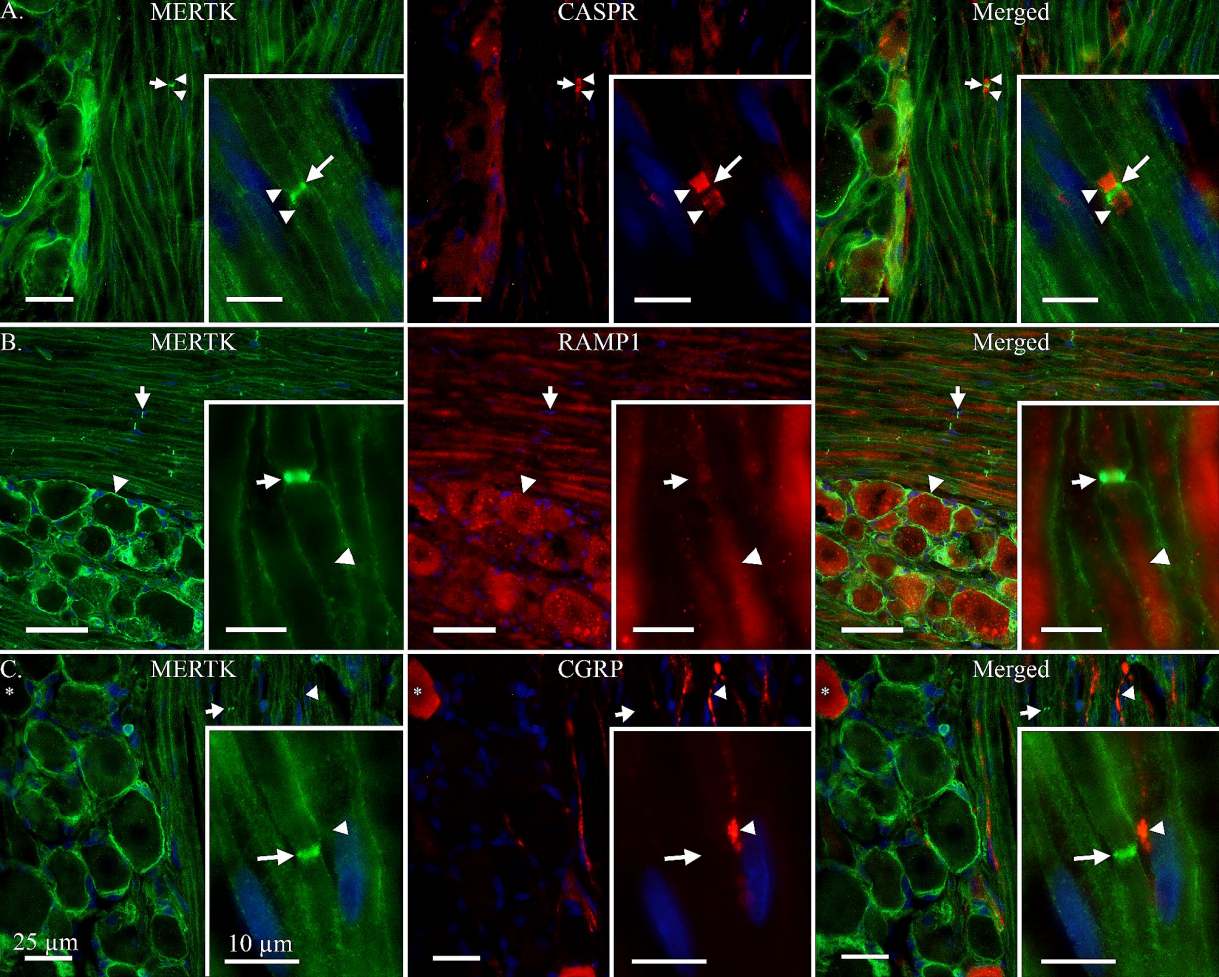
MERTK expression in relation to RAMP1, CGRP and CASPR. **A**: MER proto-oncogene tyrosine kinase (MERTK) immunoreactivity was observed mainly in satellite glial cells (SGCs) and, to a lesser extent, in Schwann cells. Interestingly, MERTK was observed to have a striking expression in the region of the Schwann cell overhanging the nodes of Ranvier of Aδ-fibres (Arrow). This was confirmed by double staining with contactin associated protein 1 (CASPR), which is explicitly expressed in the para- and juxtaparanodal regions of myelinated fibres (Arrowheads). **Insert-A**: Close-up on an Aδ-fibre with para- and juxtaparanodal regions stained by CASPR (Arrowheads). Notably, a weak expression of MERTK can be seen in the outer layer of the Schwann cell, which is strikingly pronounced where the Schwann cells border the node of Ranvier (Arrow). **B**: To confirm the expression of MERTK in Schwann cells and SGCs, a double-staining was performed with a well-established antibody, RAMP1. MERTK can be seen expressed in SGCs enveloping a RAMP1 positive neuron (Arrowhead), and a striking immunoreactivity of MERTK can be seen in Schwann cell outer membranes proximal to the nodes of Ranvier (Arrow). **Insert-B**: Close-up on an Aδ-fibre immunoreactive for RAMP1 (Arrowhead). Flanking the axon, its associated Schwann cell indicates immunoreactivity for MERTK with a strong expression near the node of Ranvier (Arrow). **C**: The expression of MERTK in relation to calcitonin gene-related peptide (CGRP). CGRP was observed in C-fibre boutons (Arrowhead) and associated neuron cell bodies (Asterix). MERTK did not co-localize with CGRP but could be observed in SGCs surrounding CGRP-positive neurons, and in Schwann cell membranes (Arrow). **Insert-C**: A C-fibre displaying immunoreactivity for CGRP with a bouton (Arrowhead) in close proximation to the MERTK-positive Schwann cell membranes flanking the node of Ranvier of an Aδ-fibre (Arrow). Inserts are separate images from the lower magnification images A, B and C

The expression of MERTK was observed mainly in SGCs enveloping neurons in the TG (Fig. 1). They can be identified by their characteristically smaller and more tightly packed nucleus, stained blue by DAPI in the figures. No immunoreactivity for MERTK was found in neuron cell bodies or Aδ-fibre axons when double-stained with RAMP1 (Fig. 1B). This was further confirmed by double staining for CGRP which was expressed in neurons and C-fibres (Fig. 1C).

To a lesser extent, MERTK was observed in the outer membrane of Schwann cells (Fig. 1). Interestingly, a markedly stronger signal was observed near the nodes of Ranvier, at the level which the Schwann cells meet. This was confirmed by double-staining with an antibody towards contactin associated protein 1 (CASPR) which marks the para- and juxtaparanodal region flanking the nodes of Ranvier (Fig. 1A). Furthermore, the MERTK signal emanating from the node of Ranvier was observed to be able to align with CGRP positive C-fibre boutons (Fig. 1C).

**Fig. 2:**
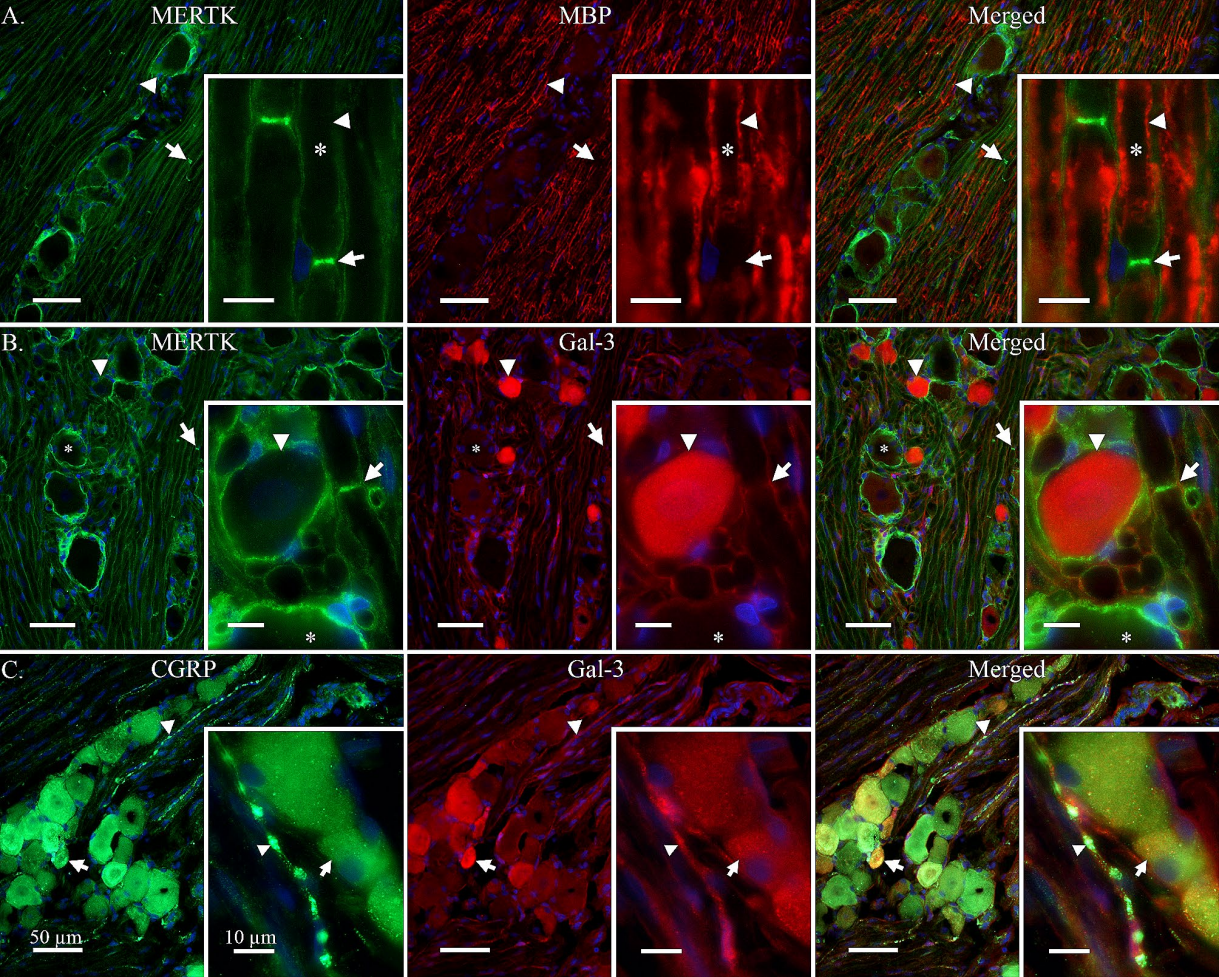
MERTK and galectin-3 expression in rat TG. **A**: To evaluate the expression of MERTK in Schwann cells enveloping Aδ-fibres, a double-staining with myelin basic protein (MBP) was performed. MBP was observed flanking the axon of A-fibres (Arrowhead) but did not co-localize with MERTK in the outer layer of Schwann cells, or the region proximal to the nodes of Ranvier (Arrow). No immunoreactivity for MBP was observed in neuron cell bodies. **Insert-A**: Close-up displaying an Aδ-fibre axon (Asterix), its associated myelin sheath stained with MBP (Arrowhead) and a prominent MERTK expression near the node of Ranvier (Arrow). **B**: MERTK expression in relation to one of its ligands, galectin-3 (Gal-3). Interestingly, Gal-3 immunoreactivity was mainly expressed in a population of smaller neurons (Arrowhead) while larger neurons often were negative (Asterix). MERTK expression was again observed in satellite glial cells (SGCs) and Schwann cells (Arrow). **Insert-B**: A Gal-3 positive neuron (Arrowhead), enveloped by MERTK positive SGCs. In comparison, a negative neuron enveloped by MERTK immunoreactive SGCs can be observed at the bottom of the picture (Asterix). The arrow points out a nearby MERTK immunoreactive Schwann cell membranes flanking a node of Ranvier. **C**: Gal-3 was double stained with CGRP to determine co-localization in smaller neurons and C-fibres. CGRP was observed in a population of neurons and associated C-fibres (Arrowhead). Co-localization with Gal-3 and CGRP was observed in some, but not all, neuron cell bodies (Arrow). **Insert-C**: A weak expression of Gal-3 co-expressed with CGRP was observed in a C-fibre bouton (Arrowhead). Similarly, a weak expression of both Gal-3 and CGRP can be observed in a nearby neuron cell body (Arrow). Inserts are separate images from the lower magnification images A, B and C

To further investigate the expression of MERTK in the Schwann cells and axons, a double-staining with myelin basic protein (MBP) was performed (Fig. 2A). This showed that only the outer membrane of the Schwann cell, as well as the region proximal to the nodes of Ranvier, displayed immunoreactivity for MERTK. While the inner layers of the Schwann cell, closer to the A-fibre axon, displayed a strong signal for myelin (Fig. 2A).

Double-staining of Gal-3 with MERTK revealed that Gal-3 was mainly expressed in a population of smaller neurons (Fig. 2B). A diffuse signal could also be observed from Schwann cells and suspected C-fibres. The Gal-3 expression in CGRP expressing neurons and C-fibres was further confirmed by double-staining with an antibody towards CGRP (Fig. 2C). Notably, the co-localization of Gal-3 and CGRP did not occur in all neurons. Furthermore, the Gal-3 signal emanating from C-fibres was considerably weaker than that coming from the population of small neurons.

### *MERTK* gene expression and ligands in patients with CH

**Fig. 3:**
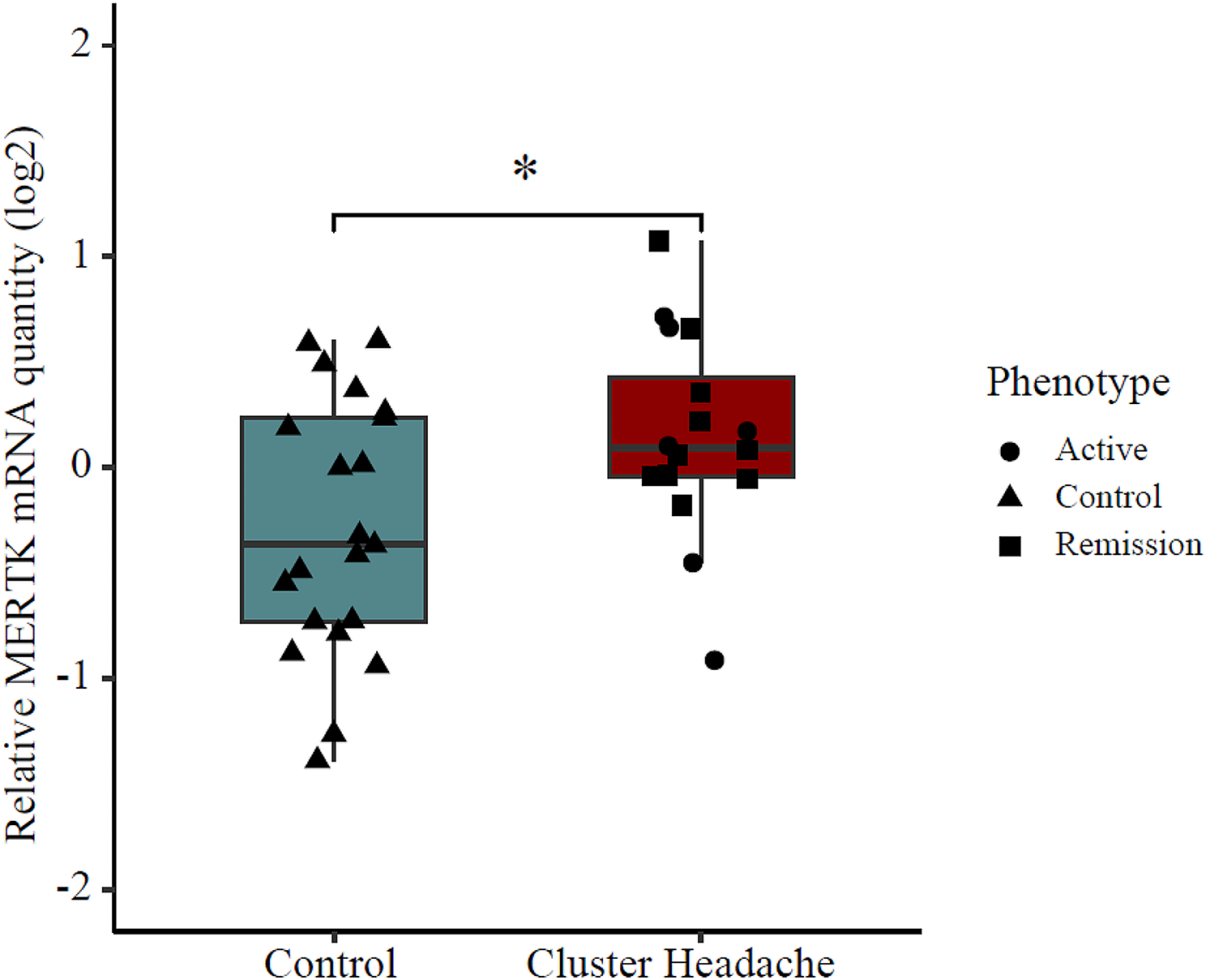
MERTK gene expression analysis in blood from cluster headache patients and controls. mRNA levels are expressed as relative quantity, levels were normalized to TBP and IPO8 and to a control sample and log2 transformed. Boxplots represent the interquartile range and median value (horizontal line), whiskers correspond to 1.5 times the interquartile range in both directions, scattered symbols show the individual values; triangles = controls, circles = patients in active bout, squares = patients in remission. Patients *n* = 16 and controls *n* = 21. Group comparison by two-tailed t-test, p-value = 0.031, *; p-value < 0.05. TBP; TATA-Box Binding Protein, IPO8; Importin 8

Gene expression of *MERTK* was analysed in blood samples from 16 study participants with episodic CH and 21 control individuals, the details of the cohort are specified in Table 1. Six of the patients were in active bout and the remaining ten patients were in remission period. Males were overrepresented in the CH group, and the average age was slightly higher (9.6 years) than in the control group (Table 1). Relative *MERTK* gene expression was analysed in patients and controls, revealing elevated *MERTK* mRNA levels in CH patients. The relative average quantity (log transformed) in CH patients was 0.15 as compared to -0.29 in controls, (t = -2.48, p-value = 0.018), (Fig. 3). Subgroup analysis further showed that *MERTK* mRNA levels were similar in CH patients in active bout to CH patients in a remission period (relative quantity 0.05 vs., 0.21, t = -0.58, p-value = 0.58).

**Fig. 4:**
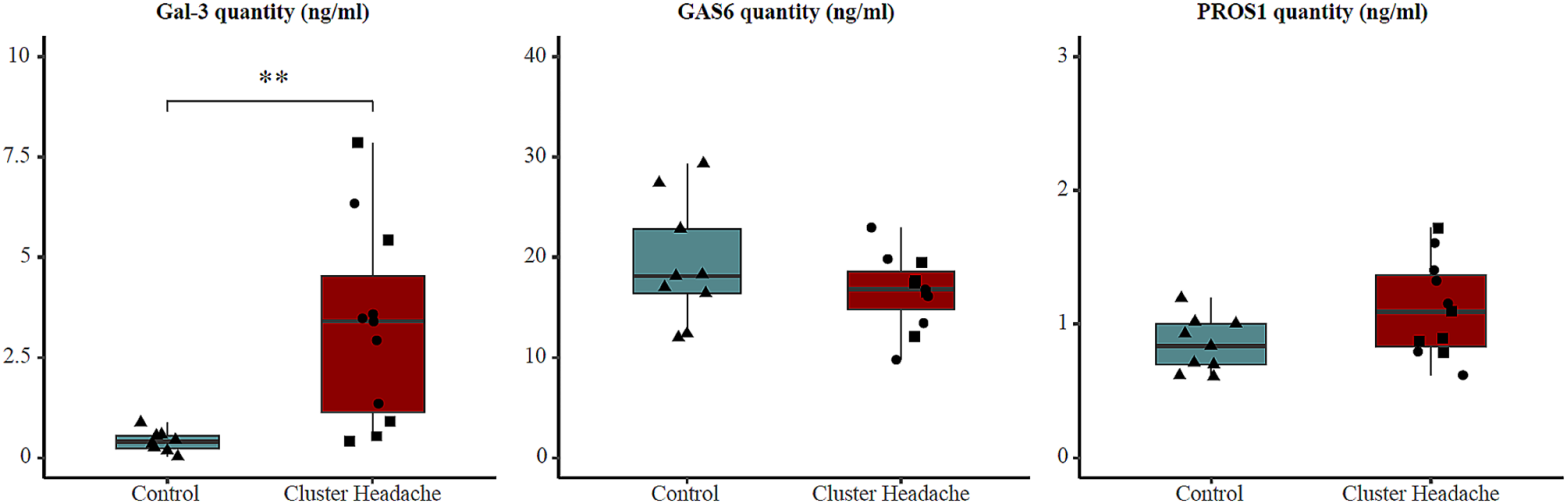
Quantification of MERTK ligands Gal-3, GAS6 and PROS1 in serum from patients and controls. Concentrations of MERTK ligands in serum expressed as ng/ml serum. Left panel: Gal-3, patients *n* = 11 and controls *n* = 8. Middle panel: GAS6, patients *n* = 11 and controls *n* = 9. PROS1, patients *n* = 11 and controls *n* = 9. Boxplots represent the interquartile range and median value (horizontal line), whiskers correspond to 1.5 times the interquartile range in both directions, scattered symbols show the individual values; triangles = controls, circles = patients in active bout, squares = patients in remission. Group comparison by Wilcoxon test or two-tailed t-test, **; p-value < 0.01

Serum samples from 11 CH patients (10 episodic and 1 chronic) and 9 controls were used for quantification of MERTK ligands Gal-3, GAS6, and PROS1. 6 of the 11 CH patients were in active bout and 5 were in a remission period (Table 1). All three analysed MERTK ligands were detectable in serum and GAS6 was the most abundant (Fig. 4). Statistical analysis revealed that Gal-3 was present at higher concentrations in serum from CH patients compared to controls, with an average concentration in patients of 3.29 ng/ml as compared to 0.41 ng/ml in controls (p-value = 0.0022, p_c_-value = 0.0067). PROS1 showed a trend of association with somewhat elevated levels in CH patients (1.11 ng/ml in patients and 0.85 ng/ml in controls), but this trend did not hold for correction for multiple testing (p-value = 0.051, p_c_-value = 0.15). GAS6 did not differ between the two groups (16.57 ng/ml in patients vs. 19.32 ng/ml in controls, p-value = 0.26). Subgroup analysis showed that in concordance with our data on *MERTK* gene expression levels, the concentration of MERTK ligands in serum did not differ between CH patients in active bout and in remission. The average concentration of Gal-3 was 3.51 ng/ml in active bout vs. 3.04 ng/ml in remission (p-value = 0.54), for GAS6 the corresponding values were 16.51 ng/ml vs. 16.64 ng/ml (p-value = 0.95), and for PROS1 1.15 ng/ml vs. 1.07 ng/ml, (p-value = 0.75).

## Discussion

We investigated *MERTK* gene expression in human tissue from CH patients and controls, comparing mRNA expression levels. Our results showed increased *MERTK* expression in tissue from CH patients in line with previous findings from GWAS studies on CH where the identified genetic markers in MERTK represent eQTLs associated with higher mRNA levels.

The MERTK ligand Gal-3 was further found to be increased in serum samples from CH patients compared to controls. Increased Gal-3 levels have previously been reported in patients with migraine [32], though the Gal-3 levels did not differ ictally from interictally. Interestingly, we do not detect any major difference in Gal-3 levels between CH patients in a remission period or during active bout. This suggests that the increase in Gal-3 might not be related to the occurrence of attacks, but rather that the increase of Gal-3 is symptomatic of the disease or related to underlying pathophysiological mechanisms. A recent study sampling CGRP plasma levels from 201 patients diagnosed with CH revealed a similarly complex relationship [36].

An important limitation of our study is the access to relevant tissues. Our investigations have been limited to blood samples because of restricted access to human nervous tissue. Moreover, the size of the patient pool is limited as CH is a rare disorder and it is difficult to identify and enroll patients into research studies during an active bout. Future validation of these findings in other tissues would be of great value.

Our results show an increase in both Gal-3 ligand and receptor expression in tissue from patients with CH suggesting increased MERTK signalling in CH pathophysiology. Although *MERTK* has not been genetically associated to migraine in GWAS [37], increased Gal-3 in migraine patients have been observed in several studies [32–34], and we hypothesize that the Gal-3 - MERTK pathway has relevance for both migraine and CH. The hypothesis is further supported by our immunohistological findings in rats, placing both Gal-3 and MERTK in disease relevant cells in the TG.

### Crosstalk

Neurons in the TG do not receive any direct synapses from other local neurons and are further separated from each other by glial cells and connective tissue [38]. However, it has been shown that crosstalk can occur in numerous ways within the TG [10].

Firstly, crosstalk between neurons and surrounding glial cells may occur in the form of increased intracellular Ca^2+^ levels in both neurons and SGCs. This occurs when the encapsulated neuron is introduced to electrical or mechanical stimulation, and similar results are obtained when instead the SGCs are stimulated [39]. Interestingly, nearby SGCs not directly affiliated to the stimulated neuron were also found to react to this by increased intracellular Ca^2+^ levels. Moreover, the coupling between SGCs and surrounding cells (both SGCs and neurons) was enhanced under experimental inflammatory conditions [40]. Furthermore, electrical ‘cross-depolarization’ has demonstrated that the depolarization of one neuron leads to activation of nearby neurons via a second messenger, assumed to be purinergic (ATP) in nature [39]. In dorsal root ganglion (DRG), ATP release has been shown to activate P2X7 on SGCs which in turn can lead to the release of tumour necrosis factor-α (TNFα) from SGCs [41]. TNFα enhances the effects of ATP on P2X3 receptors, thereby heightening the excitability of DRG neurons [41].

Other relevant ways in which crosstalk occurs in the TG include paracrine and autocrine signalling of neuropeptides, nitric oxide and inflammatory cytokines [10, 42].

We have previously shown the relationship between CGRP containing C-fibre boutons aligning with the nodes of Ranvier on proximal Aδ-fibres immunoreactive for the CGRP receptor [43]. This relationship can also be observed for Substance P and neurokinin receptors in the TG [44]. Additionally, we have suggested that the local release of CGRP from C-fibre boutons likely target CGRP receptors expressed on the Aδ-fibre axon accessible at the node of Ranvier [43].

The presence of MERTK at the perinodal gap suggests its involvement in the crosstalk relationship between the C-fibre bouton, Aδ-fibre, and proximal Schwann cells (Fig. 1). This relationship could further be characterized as a tripartite synapse [45]. The MAPK/ERK_1/2_ pathway is one of the intracellular signalling pathways activated by the MERTK receptor [24]. Interestingly, a study on rat DRG revealed that ERK_1/2_ phosphorylation in Aδ-fibre neurons and SGCs is involved in the generation of pain hypersensitivity after tissue injury, which could be abolished by the intervention of a mitogen-activated protein kinase 1/2 (MEK_1/2_) inhibitor [46]. Previous studies have shown that locally released neurotransmitters induce intracellular Ca^2+^ release in perisynaptic Schwann cells [45, 47]. The plausible co-release of Gal-3 (Fig. 2C) from C-fibre boutons could thus lead to the activation of the MERTK receptor situated on the perisynaptic Schwann cells, which in turn induces the MAPK/ERK_1/2_ pathway. Interestingly, another CH GWAS candidate gene, dual specificity phosphatase 10 (DUSP10) located on 1q41, regulates ERK_1/2_ activity by suppressing ERK-dependent gene expression [48]. This suggests that activation of the MAPK/ERK_1/2_ pathway in glial cells could play a role in the sensitization of trigeminal afferents with relevance for primary headache.

Another aspect is that in the CNS, MERTK has an important role in promoting synaptic-pruning in microglia, macrophages and astrocytes [49]. MERTK works in synergy with the Multiple EGF-like domains 10 receptor (MEGF10) responding to “eat-me” signals to engulf undesired synapses [50]. MERTK is involved in the rearrangement of the cytoskeleton required for the engulfment process [51]. Ablation of chaperone cell cycle control protein 50 A (Cdc50a) in an animal model resulted in excessive elimination of inhibitory post-synaptic synapses, leading to an overexcitation of neurons and seizure like symptoms. Cdc50a is a flippase protein whose function is to transport phosphatidylserine to the cell surface to allow it to work as an “eat-me” signal. When MERTK was ablated in the microglia of the rodent model the excessive pruning of the inhibitory synapses was aborted suggesting MERTK plays a crucial role in synapse pruning, particularly for inhibitory synapses [52].

MERTK ligands such as PROS1, GAS6, and Gal-3 function as crucial bridging molecules between the phagocyte and the target molecule to be engulfed. However, the ligands display different specificity; Gal-3 binds to exposed desialylated sugar chains such as galactose residues while PROS1 and GAS6 binds to phosphatidylserine residues [53, 54]. Interestingly, only Gal-3 induces the externalization of phosphatidylserine, though as of yet, this has only been shown in neutrophils and T-cells [55–57]. The exposure of phosphatidylserine on the cell surface is reversibly dependent on intracellular Ca^2+^ levels with high levels of Ca^2+^ leading to exposed phosphatidylserine [56]. Interestingly, the most commonly used prophylactic treatment for CH, verapamil, block L-type Ca^2+^ channels, a class of channels which typically lead to release of intracellular Ca^2+^ upon activation [58]. 

**Fig. 5:**
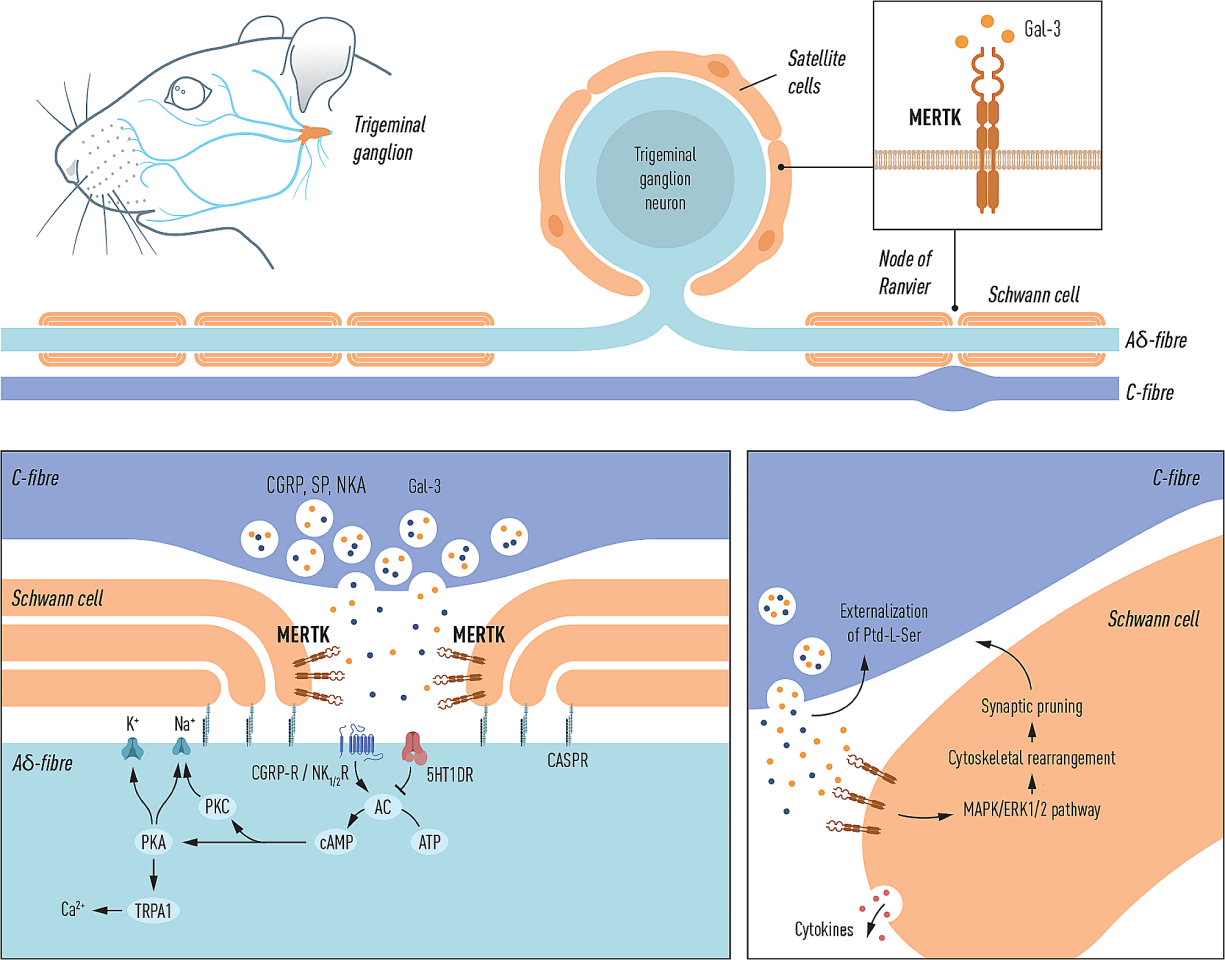
Schematic of proposed mechanism of action at the trigeminal tripartite synapse. **Top**: Pseudo-unipolar trigeminal neuron projecting a myelinated Aδ-fibre which aligns with a C-fibre bouton. MERTK receptor expression can be observed in satellite glial cells (SGCs) and Schwann cells. **Bottom-left**: Depolarization of C-fibre axons causes the local release of headache relevant neuropeptides, e.g. calcitonin gene-related peptide (CGRP), substance P (SP) and neurokinin A (NKA) [43, 44, 59], these neuropeptides could traverse the nodal gap and activate their respective receptors, expressed in the Aδ-fibre axon at the node of Ranvier, leading to modulation and/or sensitization of the nociceptive signal projecting to higher order neurons. This is suggested to occur via activation of the cAMP dependent pathway, activating protein kinase A (PKA) and C (PKC), which leads to an increase in intracellular Ca^2+^ [60]. Similarly, Gal-3 could be co-released from the C-fibre bouton and activate MERTK receptors expressed in the Schwann cell membranes flanking the node of Ranvier. **Bottom-right**: The hypothesized local release of Gal-3 from C-fibre boutons likely activates MERTK receptors in the Schwann cell membrane. We propose that this activation could lead to the activation of the MAPK/ERK_1/2_ pathway in the Schwann cell which produces hypersensitivity which can be abolished by MEK1/2 inhibitor intervention, as shown in dorsal root ganglia [46]. Finally, the release of Gal-3 can also lead to the externalization of phosphatidylserine (Ptd-L-Ser), which potentially could be a driving factor for the mechanism of synaptic pruning

If the upregulation of Gal-3 and MERTK, observed in blood of CH patients, is mirrored in the nervous tissue it could also affect the sensitization process of the trigeminal pain pathway. We suggest a compiled schematic of how this process could occur in figure 5. This is speculative and future studies are required to elucidate if Gal-3 and MERTK have a pathophysiological role in CH.

## Conclusion

This study points to a possible role for MERTK in headache pathophysiology by showing strong expression in the glial cells of the TG, and an upregulation of MERTK and its ligand Gal-3 in CH patients. MERTK is known to mediate phagocytosis, efferocytosis, and anti-inflammatory cascades. The specific MERTK staining at the perinodal gap suggests a role of MERTK in the crosstalk between glial and neuronal cells in the trigeminal ganglia and possibly in the sensitization of trigeminal afferents. It is currently unclear if the observed upregulation of MERTK and Gal-3 in CH patients is pathological, or a physiological response to the clinical symptoms of CH. However, the presence of MERTK in neurons and satellite glia cells in the rat trigeminal ganglia highlights MERTK signalling as an interesting potential target in primary headache.

### Electronic supplementary material

Below is the link to the electronic supplementary material.

Supplementary Material 1

## Data Availability

The datasets generated during and/or analyzed during the current study are available from the corresponding author on reasonable request.
