# Supplementary material for: MERTK in the rat trigeminal system: a potential novel target for cluster headache?
Source: J Headache Pain. 2024 May 23;25(1):85. doi: 10.1186/s10194-024-01791-6 (PMC11119394; doi:10.1186/s10194-024-01791-6)
Supplement: Supplementary file 1 — Supplementary Material 1 [file 10194_2024_1791_MOESM1_ESM.docx]

Table 1a. Primary antibodies used for immunohistochemistry.

| **Name (#Cat)** | **Dilution** | **Host** | **Immunogen** | **Supplier** |
| --- | --- | --- | --- | --- |
| Anti-MERTK antibody [Y323] (ab52968) | 1:100 | Rabbit | Synthetic peptide within Human MERTK aa 1-100 (N terminal). The exact sequence is  proprietary. | Abcam, Cambridge, UK |
| Anti-CGRP antibody (ab81887) | 1:100 | Mouse | Rat alpha-CGRP | Abcam, Cambridge, UK |
| Anti-CASPR, clone K65/35 (MABN69) | 1:100 | Mouse | \| Recominant protein corresponding to rat Caspr. \|  \| \| --- \| --- \| | EMD Millipore Corporation, Temecula, CA, USA. |
| Anti-MBP antibody (MA5–15922) | 1:100 | Mouse | Purified recombinant fragment of human MBP expressed in *E. coli*. | Thermo Fisher Scientific, Waltham, MA, USA |
| Anti-Galectin 3 antibody (AB278071) | 1:100 | Mouse | Recombinant full length protein corresponding to Human Galectin 3. | Abcam, Cambridge, UK |
| Anti-RAMP1 antibody  (844) | 1:200 | Goat | C-terminal of human RAMP1 | Merck & Co, Inc., West Point, PA, USA |

Table 1b. Secondary antibodies used for immunohistochemistry.

| **Conjugate** | **Dilution** | **Against** | **Supplier** |
| --- | --- | --- | --- |
| Alexa488 | 1:100 | Anti-rabbit | Thermo Scientific, IL, USA |
| Cy3 | 1:200 | Anti-goat | Jackson Immunoresearch, West Grove, PA, USA |
| Alexa594 | 1:100 | Anti-rabbit | Thermo Scientific, IL, USA |
| Alexa594 | 1:100 | Anti-mouse | Thermo Scientific, IL, USA |
| FITC | 1:150 | Anti-rabbit | Thermo Scientific, IL, USA |
